# MDS, Hermitian almost MDS, and Gilbert–Varshamov quantum codes from generalized monomial-Cartesian codes

**DOI:** 10.1007/s11128-024-04297-x

**Published:** 2024-03-01

**Authors:** Beatriz Barbero-Lucas, Fernando Hernando, Helena Martín-Cruz, Gary McGuire

**Affiliations:** 1 School of Mathematics and Statistics, University College Dublin, Dublin, Ireland; 2 Instituto Universitario de Matemáticas y Aplicaciones de Castellón and Departamento de Matemáticas, Universitat Jaume I, Campus de Riu Sec, 12071 Castelló, Spain

**Keywords:** error-correction, Hermitian, MDS, Gilbert-Varshamov

## Abstract

We construct new stabilizer quantum error-correcting codes from generalized monomial-Cartesian codes. Our construction uses an explicitly defined twist vector, and we present formulas for the minimum distance and dimension. Generalized monomial-Cartesian codes arise from polynomials in *m* variables. When $m=1$ our codes are MDS, and when $m=2$ and our lower bound for the minimum distance is 3, the codes are at least Hermitian almost MDS. For an infinite family of parameters, when $m=2$ we prove that our codes beat the Gilbert–Varshamov bound. We also present many examples of our codes that are better than any known code in the literature.

## Introduction

Certain classically intractable problems can become feasible when approached with the computational power of quantum computers. This was demonstrated through Shor’s algorithm, which solves in polynomial time the prime factorization problem and discrete logarithm problem on quantum computers [50]. Due to this fact, researchers and companies are actively engaged in constructing quantum computers with many qubits [10, 15]. Quantum computer implementations have higher error rates than classical computers, making reliability a challenge. However, despite quantum information being unclonable [18, 56], it was shown that quantum error correction techniques can be used [49, 53]. Over the last twenty-five years, error correction has proved to be one of the main obstacles to scaling up quantum computing and quantum information processing.

There is an extensive study of quantum error-correcting codes, see for example the papers [3, 4, 11, 12, 31, 33, 52] for the binary case and [5, 6, 9, 14, 21, 24, 27, 32, 35, 40, 41, 47, 51] for the general case. Many of the known quantum error-correcting codes are stabilizer codes. Let ${{\mathbb {C}}}$ be the complex field, let *q* be a prime power and let *n* be a positive integer. A stabilizer code $Q\ne \{0\}$ is the common eigenspace of an abelian subgroup of the error group $G_n$ generated by a nice error basis on the space ${{\mathbb {C}}}^{q^n}$ (see [36, 37] for details). The code *Q* has minimum distance *d* whenever all errors in $G_n$ with weight less than *d* can be detected, or have no effect on *Q*, but some errors of weight *d* cannot be detected. A code as above has parameters $[[n,k,d]]_q$ when it is a $q^k$-dimensional subspace of ${{\mathbb {C}}}^{q^n}$ and has minimum distance *d* (see, for instance, [12, 35]). Stabilizer quantum error-correcting codes have been studied by many authors because they can be constructed from classical additive codes in ${\mathbb {F}}_q^{2n}$, which are self-orthogonal with respect to a trace symplectic form. In particular, stabilizer codes can be obtained from suitable Hermitian self-orthogonal classical linear codes (see [35] or [5, 9, 12] for details). We will utilize this construction.

Many constructions of classical codes start with a quotient polynomial ring of the form ${{\mathbb {F}}}_q[X_1,\ldots ,X_m]/I$ where *I* is an ideal. Affine variety codes were introduced by Fitzgerald and Lax in [23], with a general ideal *I*. Our codes $C_{{\varvec{v}},\Delta ,Z}$ (defined in the next section) are a type of generalized affine variety code, so we could use this name. However, since the codes we define are generalized monomial-Cartesian codes, introduced in [45], and although the definition is slightly different, we are going to call our codes $C_{{\varvec{v}},\Delta ,Z}$ *generalized monomial-Cartesian codes*.

Monomial-Cartesian codes (MCCs) are a class of evaluation codes obtained as the image of maps

$$\begin{aligned} {{\,\textrm{ev}\,}}_S:V_\Delta \subset {\mathbb {F}}_q[X_1,\dots ,X_m]/I \longrightarrow {\mathbb {F}}_q^{n} \text {, } \quad {{\,\textrm{ev}\,}}_S(f)=\left( f(\varvec{\beta }_1),\dots ,f(\varvec{\beta }_n)\right) , \end{aligned}$$

where *m* is a positive integer larger than 1, $S=S_1\times \cdots \times S_m=\{\varvec{\beta }_1,\dots ,\varvec{\beta }_n\}$ is a Cartesian-product subset of ${\mathbb {F}}_q^m$, *I* is the vanishing ideal at *S* of ${\mathbb {F}}_q[X_1,\dots ,X_m]$, and $V_\Delta$ is an ${\mathbb {F}}_q$-linear space generated by classes of monomials. MCCs were introduced in [45] with only algebraic tools, see also [46]. These codes have several different applications in the literature, such as quantum codes, locally recoverable codes (LRCs) with availability, polar codes and $(r,\delta )$-LRCs [13, 26, 45].

Generalized monomial-Cartesian codes arise when changing the evaluation map ${{\,\textrm{ev}\,}}_S$ to twist each coordinate of ${{\,\textrm{ev}\,}}_S(f)$ by nonzero elements of ${{\mathbb {F}}}_q$. In this article, we will use generalized MCCs, where the set $S_1$ is a certain fixed set, and we will use the same name for this construction, see Definition 2.3. We will use generalized monomial-Cartesian codes to construct Hermitian self-orthogonal classical linear codes and thereby construct stabilizer quantum codes. We present some evidence comparing our codes to codes in [8, 14, 16, 28, 38, 43, 55], which shows that they are very good quantum codes, and sometimes optimal.

Quantum MDS codes are those achieving the quantum singleton bound; there are many papers on this type of codes. (Some recent papers are [7, 19, 42].) The MDS conjecture limits the length of a *q*-ary quantum MDS code to be at most $q^2+2$ [35]. Thus, another goal is to obtain longer *q*-ary codes with good parameters. With our construction, we achieve this.

The paper is laid out as follows: After the preliminaries in Sect. 2, we present our construction in Sect. 3. Previous works using a twist vector have proved the existence of a twist vector with the required properties, whereas a feature of our construction is that we define the twist vector explicitly, see (3) in Sect. 3. We present a general construction first (Theorem 3.4) and then a more specific construction that allows us to control the minimum distance (Theorem 3.7). In Sect. 4, we will show that our construction with $m=1$ gives MDS codes. We also prove that when $m=2$ and our lower bound for the minimum distance is 3 the codes are at least Hermitian almost MDS. Section 5 contains a proof that for an infinite family of parameters when $m=2$, our codes beat the Gilbert–Varshamov bound. Finally, in Sect. 6 we present some examples with small parameters that beat the best known codes in the literature.

## Preliminaries

In this paper, we will assume that *q* is odd, although in this section the definitions hold for any *q*. Let us denote by ${{\mathbb {N}}}$ the set of positive integers and by ${{\mathbb {N}}}_0$ the set of nonnegative integers. For any two vectors ${\varvec{a}}=(a_0,\dots ,a_{n-1})$, ${\varvec{b}}=(b_0,\dots ,b_{n-1}) \in {{\mathbb {F}}}_{q^2}^n$, their Hermitian inner product is defined as:

$$\begin{aligned}{\varvec{a}} \cdot _h {\varvec{b}}=\sum _{i=0}^{n-1} a_ib_i^q,\end{aligned}$$

their Euclidean inner product is defined as:

$$\begin{aligned}{\varvec{a}} \cdot _e {\varvec{b}}=\sum _{i=0}^{n-1} a_ib_i,\end{aligned}$$

and their * product is defined as:

$$\begin{aligned}(a_0,\dots ,a_{n-1})*(b_0,\dots ,b_{n-1})=(a_0\cdot b_0,\dots ,a_{n-1}\cdot b_{n-1}).\end{aligned}$$

Let the symbol $\perp _h$ (respectively, $\perp _e$) mean dual with respect to Hermitian (respectively, Euclidean) inner product. For a vector subspace (or code) *C* of ${{\mathbb {F}}}_{q^2}^n$, we let $C^{\perp _h}$ (respectively, $C^{\perp _e}$) denote the orthogonal vector subspace (the dual code) with respect to the Hermitian (respectively, Euclidean) inner product. We denote by ${{\,\textrm{d}\,}}(C)$ the minimum distance of *C*. Let *s* be a nonnegative integer and ${\varvec{c}}=(c_0,\dots ,c_{n-1})\in C$ be a codeword. We denote ${\varvec{c}}^{s}=(c_0^s,\dots ,c_{n-1}^s)$ and

$$\begin{aligned}C^s:=\{{\varvec{c}}^s \mid {\varvec{c}}\in C\}\subseteq {{\mathbb {F}}}_{q^2}^n.\end{aligned}$$

Let us denote by ${{\,\textrm{w}\,}}({\varvec{c}})$ the Hamming weight of ${\varvec{c}}$. We say that two codes are isometric if there exists a bijective mapping between them that preserves Hamming weights.

### Theorem 2.1

([1, 35]) Let *C* be a linear [*n*, *k*, *d*] error-correcting code over the field ${{\mathbb {F}}}_{q^2}$ such that $C\subseteq C^{\perp _h}$. Then, there exists an $[[n,n-2k,\ge d^{\perp _h}]]_q$ stabilizer quantum code, where $d^{\perp _h}$ stands for the minimum distance of $C^{\perp _h}$.

The idea in this paper is to construct codes that satisfy the hypotheses of Theorem 2.1. In order to do so, we fix a finite field ${{\mathbb {F}}}_{q^2}$. Let ${{\mathbb {F}}}_{q^2}[X_1,\dots ,X_m]$ be the polynomial ring in $m\ge 1$ variables over ${{\mathbb {F}}}_{q^2}$. For each element ${\varvec{e}}=(e_1,\dots ,e_m)\in {{\mathbb {N}}}_0^m$, we write $X^{{\varvec{e}}}$ for $X_1^{e_1}X_2^{e_2}\cdots X_m^{e_m}$. We will refer to ${\varvec{e}}$ as an exponent and use the lexicographic order in ${{\mathbb {N}}}_0^m$ for the exponents. That is, given ${\varvec{e}}$, $\varvec{e'}\in {{\mathbb {N}}}_0^m$, we say ${\varvec{e}}<\varvec{e'}$ if and only if $e_1<e'_1$ or there exists $j\in \{2,\dots , m\}$ such that $e_1 =e'_1, \dots , e_{j-1} = e'_{j-1}$ and $e_j<e'_j$. Any order can be used.

Let $\lambda \in {{\mathbb {N}}}$ such that $\lambda \mid q-1$. Let $A_1$ be the set of roots of the polynomial $X_1^{\lambda (q+1)}-1$, which lie in ${{\mathbb {F}}}_{q^2}$. We also consider arbitrary subsets $A_j \subseteq {{\mathbb {F}}}_{q^2}^*$ for $j=2, \dots , m$ which have cardinality greater than or equal to 2. Let $a_j :=\# A_j$ for $j=1, \ldots , m$, so that $a_1=\lambda (q+1)$. Let

$$\begin{aligned} Z :=A_1 \times \cdots \times A_m, \end{aligned}$$

which has cardinality

$$\begin{aligned}n:=\prod _{j=1}^m a_j.\end{aligned}$$

Let

$$\begin{aligned}Q_j(X_j)=\prod _{\beta \in A_j}(X_j - \beta )\end{aligned}$$

be the monic polynomial in one variable whose roots are the elements of $A_j$, then $\deg (Q_j)= a_j$ for $j=1,\dots ,m$. Let *I* be the ideal of ${{\mathbb {F}}}_{q^2}[X_1,\dots ,X_m]$ generated by the polynomials $Q_1(X_1)=X_1^{\lambda (q+1)}-1$ and $Q_j(X_j)$ for $j=2, \ldots , m$. Letand let

1 $$\begin{aligned} E:=\{0,1,\dots ,a_1-1\}\times \cdots \times \{0,1,\dots ,a_m-1\}. \end{aligned}$$

Given $f\in R$, in this paper *f* is going to denote both the equivalence class in *R* and the unique polynomial representing *f* in ${{\mathbb {F}}}_{q^2}[X_1,\dots ,X_m]$ with degree in $X_j$ less than $a_j$, $1\le j \le m$. Thus, one can write any $f\in R$ uniquely as

$$\begin{aligned}f(X_1,\dots ,X_m)=\sum _{(e_1,\dots ,e_m)\in E} f_{e_1,\dots ,e_m}X_1^{e_1}\cdots X_m^{e_m},\end{aligned}$$

with $f_{e_1,\dots ,e_m}\in {{\mathbb {F}}}_{q^2}$. Let us denote ${{\,\textrm{supp}\,}}(f)=\{(e_1,\dots ,e_m)\in E \mid f_{e_1,\dots ,e_m}\ne 0\}$.

### Definition 2.2

Let *E* be as defined earlier in (1). For each nonempty subset $\Delta \subseteq E$, define $V_\Delta :=\{f\in R \mid {{\,\textrm{supp}\,}}(f)\subseteq \Delta \}$.

Note that $V_\Delta$ is the ${\mathbb {F}}_{q^2}$-vector space consisting of the ${\mathbb {F}}_{q^2}$-span of $\{ X^{{\varvec{e}}} \mid {\varvec{e}}\in \Delta \}$.

For any positive integer *t*, we denote by $\zeta _t$ a primitive *t*-th root of unity. Since $A_j$ has $a_j$ elements, we choose a bijection between $A_j$ and the set $\{0,1, \dots , a_j-1\}$, and this is going to give us an ordering of $A_j$, $j=2,\dots ,m$. Let us represent by $\xi _{(j,s)}$ the elements of each set $A_j$, where the subindex $s\in \{0,1, \dots , a_j-1\}$ is given by the ordering. For $\varvec{\alpha } = (\alpha _1, \dots , \alpha _m) \in E$, we define $\varvec{P_{\alpha }} \in Z$ by

$$\begin{aligned} \varvec{P_{\alpha }}:=(\zeta _{\lambda (q+1)}^{\alpha _1}, \xi _{(1,\alpha _2)}, \dots \xi _{(m,\alpha _m)}), \end{aligned}$$

where $\alpha _1$ indicates the exponent of $\zeta _{\lambda (q+1)}$ and $\alpha _j \in \{0,1, \ldots ,a_j-1\}$ gives the position of the element $\xi _{(j,\alpha _j)}\in A_j$ in the ordering of $A_j$, $j=2,\dots ,m$. Every element of *Z* has the form $\varvec{P_{\alpha }}$ for some $\varvec{\alpha }\in E$. This sets up a bijection between *Z* and *E*.

We order the set *Z* using the (lexicographic) order in ${{\mathbb {N}}}_0^m$ restricted to *E*. That is, given $\varvec{P_\alpha }$, $\varvec{P_{\alpha '}} \in Z$, then $\varvec{P_\alpha } < \varvec{P_{\alpha '}}$ if and only if $\varvec{\alpha }<\varvec{\alpha '}$. Then, we can rename the points in *Z* as

$$\begin{aligned}\varvec{P_0}:= {\varvec{P}}_{(0,\dots ,0)}, \varvec{P_1}:= {\varvec{P}}_{(0,\dots ,0,1)}, \dots , \varvec{P_{n-1}}:= {\varvec{P}}_{(a_1-1,a_2-1,\dots ,a_m-1)}.\end{aligned}$$

Let ${\varvec{v}}=(v_0,\dots ,v_{n-1})\in ({{\mathbb {F}}}^*_{q^2})^{n}$, we will refer to this vector as the *twist vector*. We index the coordinates of ${\varvec{v}}$ by the elements of *E*, and we order the coordinates of ${\varvec{v}}$ in the same way as we ordered the elements of *Z*. That is,

$$\begin{aligned}v_0:=v_{(0,\dots ,0)}, v_1:= v_{(0,\dots ,0,1)}, \dots , v_{n-1}:= v_{(a_1-1,a_2-1,\dots ,a_m-1)}.\end{aligned}$$

The linear evaluation map in *Z*:

$$\begin{aligned} {{\,\textrm{ev}\,}}_{{\varvec{v}},Z}:R \longrightarrow {{\mathbb {F}}}_{q^2}^{n} \text {, } \quad {{\,\textrm{ev}\,}}_{{\varvec{v}},Z}(f)=\left( v_0 f(\varvec{P_0}),\dots ,v_{n-1}f(\varvec{P_{n-1}})\right) \end{aligned}$$

is injective by the definition of *R*. It provides the following class of evaluation codes.

### Definition 2.3

Let $V_\Delta$ be as defined in Definition 2.2. The *generalized monomial-Cartesian code* (*GMCC*) $C_{{\varvec{v}},\Delta ,Z}$ is the image of $V_\Delta$ via the evaluation map ${{\,\textrm{ev}\,}}_{{\varvec{v}},Z}$, that is,

$$\begin{aligned}C_{{\varvec{v}},\Delta ,Z}:={{\,\textrm{ev}\,}}_{{\varvec{v}},Z}(V_\Delta )={{\,\textrm{span}\,}}\{{{\,\textrm{ev}\,}}_{{\varvec{v}},Z}(X^{{\varvec{e}}}) \mid {\varvec{e}}\in \Delta \} \subseteq {\mathbb {F}}_{q^2}^{n}.\end{aligned}$$

Since the order of the set *Z* will be fixed for the rest of the article, we will use the notation ${{\,\textrm{ev}\,}}_{{\varvec{v}}} :={{\,\textrm{ev}\,}}_{{\varvec{v}},Z}$ and $C_{{\varvec{v}},\Delta }:=C_{{\varvec{v}},\Delta , Z}$.

### Remark 2.4

Evaluation maps of our codes are defined on subsets of coordinate rings of certain affine varieties, but these codes can also be introduced with algebraic tools. Monomial-Cartesian codes were introduced in [45] using only algebraic tools. When the set $A_1\subseteq {\mathbb {F}}_{q^2}$ is arbitrary, GMCCs extend monomial-Cartesian codes. This should be the accurate definition, but for our purposes in this paper we use this particular set $A_1$, namely the $\lambda (q+1)$-th roots of unity.

Here is a standard fact, that the dual of a GMCC is another GMCC.

### Lemma 2.5

The dual code $(C_{{\varvec{v}},\Delta })^{\perp _h}$ is a GMCC $C_{{\varvec{w}},\Delta }$ for some twist vector ${\varvec{w}}$.

### Proof

Consider any two codewords ${\varvec{c}}=(c_0,\ldots ,c_{n-1})\in C_{{\varvec{1}},\Delta }$ and ${\varvec{b}}=(b_0,\ldots ,b_{n-1})\in (C_{{\varvec{1}},\Delta })^{\perp _h}$. Then, the following equation holds:

2 $$\begin{aligned} c_0b_0^q+\cdots +c_{n-1}b_{n-1}^q=0. \end{aligned}$$

Let ${\varvec{v}}=(v_0,\dots ,v_{n-1})$ be a (fixed) vector in $({{\mathbb {F}}}^*_{q^2})^{n}$ and consider $C_{{\varvec{v}},\Delta }$. We know that ${\varvec{v}}*{\varvec{c}}=(v_0c_0,\ldots ,v_{n-1}c_{n-1})\in C_{{\varvec{v}},\Delta }$ whenever ${\varvec{c}}=(c_0,\ldots ,c_{n-1})\in C_{{\varvec{1}},\Delta }$, because

$$\begin{aligned}C_{{\varvec{1}},\Delta } \longrightarrow C_{{\varvec{v}},\Delta }, \quad {\varvec{c}}\mapsto {\varvec{v}}*{\varvec{c}}\end{aligned}$$

is a bijective mapping. We use this presentation of $C_{{\varvec{v}},\Delta }$.

We will prove that $(C_{{\varvec{v}},\Delta })^{\perp _h}=C_{{\varvec{w}},\Delta }$ where ${\varvec{w}}=(w_0,\dots ,w_{n-1})$ is defined by $w_i:=\frac{1}{v_i^q}$ for all $i=0,\ldots ,n-1$.

First we claim that for any ${\varvec{b}}\in (C_{{\varvec{1}},\Delta })^{\perp _h}$ we have that ${\varvec{w}}*{\varvec{b}}=(w_0b_0,\ldots ,w_{n-1}b_{n-1})\in (C_{{\varvec{v}},\Delta })^{\perp _h}$. To see this, choose ${\varvec{v}}*{\varvec{c}}\in C_{{\varvec{v}},\Delta }$ and note that

$$\begin{aligned} v_0c_0w_0^q b_0^q+\cdots +v_{n-1}c_{n-1}w_{n-1}^q b_{n-1}^q=0 \end{aligned}$$

using the fact that $w_i^q=1/v_i^{q^2}=1/v_i$ for all *i*, and using (2). This shows that all the vectors ${\varvec{w}}*{\varvec{b}}$ are in $(C_{{\varvec{v}},\Delta })^{\perp _h}$.

Finally note that

$$\begin{aligned}(C_{{\varvec{1}},\Delta })^{\perp _h} \longrightarrow (C_{{\varvec{v}},\Delta })^{\perp _h}, \quad {\varvec{b}}\mapsto {\varvec{w}}*{\varvec{b}}\end{aligned}$$

is a bijective mapping, which shows that $(C_{{\varvec{v}},\Delta })^{\perp _h}=C_{{\varvec{w}},\Delta }$. $\square$

The length and the dimension of a GMCC are *n* and $\#\Delta$, respectively. A bound for the minimum distance is provided in Corollary 2.8.

### Lemma 2.6

The GMCCs $C_{{\varvec{1}},\Delta }$ and $C_{{\varvec{v}},\Delta }$ are isometric.

### Proof

For any codeword ${\varvec{c}}=(c_0,\ldots ,c_{n-1})\in C_{{\varvec{1}},\Delta }$, its twisted analogue codeword ${\varvec{v}}*{\varvec{c}}=(v_0c_0,\ldots ,v_{n-1}c_{n-1})\in C_{{\varvec{v}},\Delta }$ under the bijective mapping $C_{{\varvec{1}},\Delta } \rightarrow C_{{\varvec{v}},\Delta }$, ${\varvec{c}}\mapsto {\varvec{v}}*{\varvec{c}}$ has the same Hamming weight, this is because $v_i\ne 0$ for all $i=1,\dots ,n$. $\square$

Affine variety codes admit a bound on the minimum distance, known as the footprint bound [29]. Monomial-Cartesian codes $C_{{\varvec{1}},\Delta }$ in the sense of our Definition 2.3 (the evaluation map is defined over the coordinate ring of some affine variety) are affine variety codes. This fact and Lemma 2.6 prove the next lemma, stating that this bound is also valid for GMCCs. For every exponent ${\varvec{e}}\in E$, we define

$$\begin{aligned}{{\,\textrm{D}\,}}({\varvec{e}}):=\prod _{j=1}^m (a_j-e_j).\end{aligned}$$

### Lemma 2.7

Let $C_{{\varvec{v}},\Delta }$ be a GMCC and let ${\varvec{c}}={{\,\textrm{ev}\,}}_{{\varvec{v}}}(f)\in C_{{\varvec{v}},\Delta }$ be a codeword, $f\in R$. Fix a monomial ordering on $({{\mathbb {N}}}_0)^m$ and let $X^{{\varvec{e}}}$ be the leading monomial of *f*. Then, ${{\,\textrm{w}\,}}({\varvec{c}})\ge {{\,\textrm{D}\,}}({\varvec{e}})$.

### Corollary 2.8

Let $C_{{\varvec{v}},\Delta }$ be a GMCC and let *d* be its minimum distance. Define $d_0=d_0\left( C_{{\varvec{v}},\Delta }\right) :=\min \{{{\,\textrm{D}\,}}({\varvec{e}}) \mid {\varvec{e}}\in \Delta \}$. Then, $d\ge d_0$.

### Remark 2.9

Affine variety codes were introduced in [23] for any ideal *I*. A classical result coming from the theory of Gröbner basis [17] implies that $d\ge d_0$, where *d* stands for the minimum distance of an affine variety code and $d_0$ is the cited footprint bound [29]. Independently, inspired by the algebraic geometric codes [34] the so-called Feng–Rao bound for the minimum distance of the dual code is derived [20]. It is known that every linear code is an algebraic geometric code. A similar bound (Andersen–Geil) was also given for an algebraic geometric code [2]. It turns out that for monomial-Cartesian codes the footprint bound applied to the dual code and the Feng–Rao bound coincide [25]. Although the footprint bound is more natural for the primal code, and the Feng–Rao bound is more natural for the dual code, we will always refer to them as $d_0$.

**Fig. 1:**
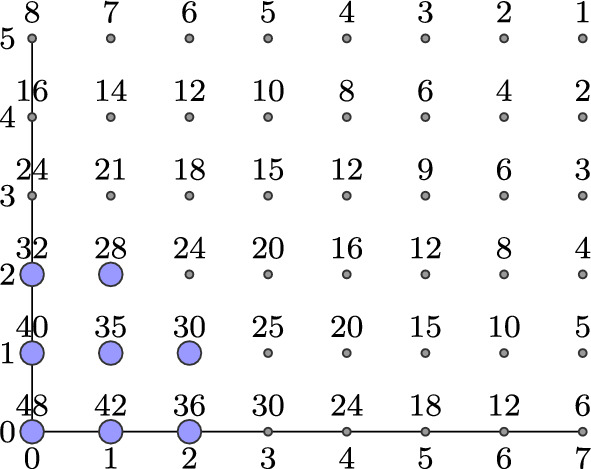
In the case $m=2$, we can use a grid to represent the set *E* so that an exponent ${\varvec{e}}=(e_1,e_2)\in E$ corresponds to the point with coordinates $(e_1,e_2)$ in the grid and that point is labelled with the integer ${{\,\textrm{D}\,}}({\varvec{e}})$. Exponents in the set $\Delta \subseteq E$ are coloured in blue. This example shows the grid representation of *E*, where $a_1=8$, $a_2=6$, and $\Delta =\left( \{0,1,2\}\times \{0,1\}\right) \cup \{(0,2),(1,2)\}$. In this example, the lower bound for the minimum distance of the code $C_{{\varvec{v}},\Delta }$ for any ${\varvec{v}}\in ({{\mathbb {F}}}_{q^2}^*)^n$ is $d_0\left( C_{{\varvec{v}},\Delta }\right) =\min \{{{\,\textrm{D}\,}}({\varvec{e}}) \mid {\varvec{e}}\in \Delta \} = 28$ by Corollary 2.8

### Lemma 2.10

Let $C_{{\varvec{v}},\Delta }$ be a GMCC. Then, $(C_{{\varvec{v}},\Delta })^{\perp _h}$ and $(C_{{\varvec{v}},\Delta })^{\perp _e}$ are isometric.

### Proof

It is straightforward because $(C_{{\varvec{v}},\Delta })^{\perp _h}=((C_{{\varvec{v}},\Delta })^{\perp _e})^q$. $\square$

### Lemma 2.11

Let $C_{{\varvec{v}},\Delta }$ be a GMCC. Then $(C_{{\varvec{1}},\Delta })^{\perp _h}$ and $(C_{{\varvec{v}},\Delta })^{\perp _h}$ are isometric.

### Proof

It follows from the fact that the family of GMCCs is closed under duality by Lemma 2.5 and by Lemma 2.6. $\square$

### Corollary 2.12

Let $C_{{\varvec{v}},\Delta }$ be a GMCC. Then $d((C_{{\varvec{v}},\Delta })^{\perp _h})=d((C_{{\varvec{1}},\Delta })^{\perp _e})$.

### Proof

This is because $(C_{{\varvec{v}},\Delta })^{\perp _h}$ and $(C_{{\varvec{1}},\Delta })^{\perp _h}$ are isometric (by Lemma 2.11) and also $(C_{{\varvec{1}},\Delta })^{\perp _h}$ is isometric to $(C_{{\varvec{1}},\Delta })^{\perp _e}$ (by Lemma 2.10). $\square$

## Stabilizer quantum codes from generalized monomial-Cartesian codes

In the present section, we construct stabilizer quantum codes by applying Theorem 2.1 to GMCCs (Definition 2.3) with a specific twist vector. Recall from Sect. 2 that *q* is an odd prime power, $\zeta _{q^2-1}$ denotes a primitive $q^2-1$-th root of unity, $\lambda \in {{\mathbb {N}}}$ is such that $\lambda \mid q-1$, $a_1= \lambda (q+1)$, $2 \le a_j \le q^2-1$ for all $j=2, \dots , m$, and $n=a_1a_2\cdots a_m$. We are going to choose the twist vector defined explicitly as follows:

3 $$\begin{aligned} {\varvec{v}}=(\underbrace{\zeta _{q^2-1}^{\frac{q-1}{2}},\dots ,\zeta _{q^2-1}^{\frac{q-1}{2}}}_{\frac{n}{q+1}}, \underbrace{1,\dots , 1}_{\frac{n}{q+1}}, \underbrace{\zeta _{q^2-1}^{\frac{q-1}{2}}, \dots ,\zeta _{q^2-1}^{\frac{q-1}{2}}}_{\frac{n}{q+1}}, \dots , \underbrace{1,\dots , 1}_{\frac{n}{q+1}})\in ({{\mathbb {F}}}_{q^2}^*)^n. \end{aligned}$$

Because

$$\begin{aligned}\left( \zeta _{q^2-1}^{\frac{q-1}{2}}\right) ^{q+1}= \zeta _{q^2-1}^{\frac{(q+1)(q-1)}{2}} = \zeta _{q^2-1}^{\frac{q^2-1}{2}}=-1\end{aligned}$$

it follows that

$$\begin{aligned} {\varvec{v}}^{q+1} = (\underbrace{-1, \dots , -1}_{\frac{n}{q+1}}, \underbrace{1, \dots , 1}_{\frac{n}{q+1}}, \underbrace{-1, \dots , -1}_{\frac{n}{q+1}}, \dots , \underbrace{1, \dots , 1}_{\frac{n}{q+1}}). \end{aligned}$$

Observe that there are $q+1$ blocks of $-1$’s or 1’s. Recall that the coordinates $v_{\varvec{\alpha }}$ of ${\varvec{v}}$ are labelled and ordered in the same way as the points $\varvec{P_\alpha }\in Z$. This twist vector works as follows. For each $\varvec{\alpha }\in E$,

4 $$\begin{aligned} v_{\varvec{\alpha }}^{q+1}= {\left\{ \begin{array}{ll} -1 &{} \text { if } 0 \le (\alpha _1 \bmod 2\lambda ) \le \lambda -1,\\ 1 &{} \text { if } \lambda \le (\alpha _1 \bmod 2\lambda ) \le 2\lambda -1. \end{array}\right. } \end{aligned}$$

Notice that $v_{\varvec{\alpha }}$ only depends on $\alpha _1$. The reason why we choose this specific twist vector is going to become clear in Proposition 3.1.

### Self-orthogonality conditions

First we present some conditions for the evaluation vectors of monomials in *R* to be orthogonal for the Hermitian inner product, when our twist vector is used.

#### Proposition 3.1

Keep the same notations as before. Let *q* be an odd prime power and consider the twist vector ${\varvec{v}}$ defined in (3). Let ${\varvec{e}}=(e_1, \dots , e_m)$, $\varvec{e'}=(e'_1, \dots , e'_m) \in E$ be exponents of two monomials $X^{{\varvec{e}}}$, $X^{\varvec{e'}} \in R$. Then, the evaluation vectors under the map ${{\,\textrm{ev}\,}}_{{\varvec{v}}}$ of these monomials are orthogonal for the Hermitian inner product if one of the following conditions hold:

- $$e_1 \equiv e'_1 \mod q+1$$, or
- $$e_1 \not \equiv e'_1 \mod \frac{q+1}{2}$$.

#### Proof

In order to compute some conditions under which two evaluations of monomials of the quotient ring *R* are orthogonal for the Hermitian inner product, we have to see when the following sum vanishes:

$$\begin{aligned} {{\,\textrm{ev}\,}}_{{\varvec{v}}}(X^{{\varvec{e}}}) \cdot _h {{\,\textrm{ev}\,}}_{{\varvec{v}}}(X^{\varvec{e'}})= \sum _{\varvec{\alpha }\in E} v_{\varvec{\alpha }}^{q+1} \zeta _{\lambda (q+1)}^{\alpha _1(e_1 + q e'_1)} \xi _{(2,\alpha _2)}^{(e_2 +q e'_2)} \cdots \xi _{(m,\alpha _m)}^{(e_m + q e'_m)}. \end{aligned}$$

Since $v_{\varvec{\alpha }}$ only depends on $\alpha _1$, we can denote by $v_{\alpha _1}:=v_{(\alpha _1, \dots , \alpha _m)}=v_{\varvec{\alpha }}$ and reorder the above sum in the following way:

$$\begin{aligned} {{\,\textrm{ev}\,}}_{{\varvec{v}}}(X^{{\varvec{e}}})\ \cdot _h \ {{\,\textrm{ev}\,}}_{{\varvec{v}}}(X^{\varvec{e'}})= & {} \left( \sum _{\alpha _1=0}^{\lambda (q+1)-1} v_{\alpha _1}^{q+1} \zeta _{\lambda (q+1)}^{\alpha _1(e_1 + q e'_1)} \right) \left( \sum _{\alpha _2=0}^{a_2-1} \xi _{(2,\alpha _2)}^{(e_2 +q e'_2)}\right) \\{} & {} \dots \left( \sum _{\alpha _m=0}^{a_m-1} \xi _{(m,\alpha _m)}^{(e_m + q e'_m)}\right) . \end{aligned}$$

We can do that because all the coordinates $v_{\varvec{\alpha }}$ in ${\varvec{v}}$ that have the same $\alpha _1$ have the same value. Now we study when the first factor equals 0, and we will ignore the other factors, since the first one gives enough information for the proof. Consider then

5 $$\begin{aligned} \sum _{\alpha _1=0}^{\lambda (q+1)-1} v_{\alpha _1}^{q+1} \zeta _{\lambda (q+1)}^{\alpha _1(e_1 + q e'_1)}, \end{aligned}$$

which is a sum over $\alpha _1\in \{0,1,\dots ,\lambda (q+1)-1\}$. We write each $\alpha _1$ in the form $k\lambda +r$ where $0\le k \le q$ and $0\le r <\lambda$. Using this to break (5) into $\lambda$ blocks of size $q+1$, using the fact that $\zeta _{q+1}:=\zeta _{\lambda (q+1)}^\lambda$ is a primitive $q+1$-th root of unity and using the structure of the twist vector ${\varvec{v}}$, we can write (5) as

$$\begin{aligned} \sum _{\alpha _1=0}^{\lambda (q+1)-1} v_{\alpha _1}^{q+1} \zeta _{\lambda (q+1)}^{\alpha _1(e_1 + q e'_1)}&= \sum _{\begin{array}{c} 0\le k\le q \\ 0 \le r < \lambda \end{array}} v_{k\lambda +r}^{q+1} \zeta _{\lambda (q+1)}^{(k\lambda +r)(e_1+qe'_1)}\\&= \sum _{k=0}^q v_{k\lambda }^{q+1} \zeta _{q+1}^{k(e_1+qe'_1)} \\&\quad + \zeta _{\lambda (q+1)}^{e_1+qe'_1} \sum _{k=0}^q v_{k\lambda +1}^{q+1} \zeta _{q+1}^{k(e_1+qe'_1)} \\&\quad + \cdots + \zeta _{\lambda (q+1)}^{(\lambda -1)(e_1+qe'_1)} \sum _{k=0}^q v_{k\lambda +\lambda -1}^{q+1} \zeta _{q+1}^{k(e_1+qe'_1)} \\&=\left( 1 + \zeta _{\lambda (q+1)}^{(e_1+qe'_1)} + \dots + \zeta _{\lambda (q+1)}^{(\lambda -1)(e_1+qe'_1)}\right) \\&\quad \left( \sum _{k=0}^{q} v_{k\lambda }^{q+1} \zeta _{q+1}^{k(e_1 + q e'_1)}\right) . \end{aligned}$$

Notice that we can do that because from (4) and the fact that $1\le \lambda \le q-1$ we have that $v_{k\lambda }^{q+1}=v_{k\lambda +1}^{q+1}=\cdots =v_{k\lambda +\lambda -1}^{q+1}$ for all $0\le k \le q$. Now using again (4) and the fact that $\zeta _{\frac{q+1}{2}}:=\zeta _{q+1}^2$ is a primitive $\frac{q+1}{2}$-th root of unity, we rewrite the last sum in the following way:

$$\begin{aligned} \sum _{k=0}^{q} v_{k\lambda }^{q+1} \zeta _{q+1}^{k(e_1 + q e'_1)}&= \sum _{k=0}^{\frac{q-1}{2}} v_{2k\lambda }^{q+1} \zeta _{q+1}^{2k(e_1 + q e'_1)}+\sum _{k=0}^{\frac{q-1}{2}} v_{2k\lambda +1}^{q+1} \zeta _{q+1}^{(2k+1)(e_1 + q e'_1)}\\&=\sum _{k=0}^{\frac{q-1}{2}} v_{2k\lambda }^{q+1} \zeta _{q+1}^{2k(e_1 + q e'_1)}-\zeta _{q+1}^{e_1+qe'_1} \sum _{k=0}^{\frac{q-1}{2}} v_{2k\lambda }^{q+1} \zeta _{q+1}^{2k(e_1 + q e'_1)}\\&=\zeta _{q+1}^{e_1+qe'_1}\left( \sum _{k=0}^{\frac{q-1}{2}} \zeta _{\frac{q+1}{2}}^{k(e_1 + q e'_1)}\right) -\left( \sum _{k=0}^{\frac{q-1}{2}} \zeta _{\frac{q+1}{2}}^{k(e_1 + q e'_1)}\right) \\&= (\zeta _{q+1}^{e_1+qe'_1}-1)\left( \sum _{k=0}^{\frac{q-1}{2}} \zeta _{\frac{q+1}{2}}^{k(e_1 + q e'_1)}\right) . \end{aligned}$$

Thus, we have shown that we can write (5) as

$$\begin{aligned} \sum _{\alpha _1=0}^{\lambda (q+1)-1} v_{\alpha _1}^{q+1} \zeta _{\lambda (q+1)}^{\alpha _1(e_1 + q e'_1)}=P\left( \zeta _{\lambda (q+1)}^{e_1+qe'_1}\right) \biggl (\zeta _{q+1}^{e_1+qe'_1}-1\biggr )\left( \sum _{k=0}^{\frac{q-1}{2}} \zeta _{\frac{q+1}{2}}^{k(e_1 + q e'_1)}\right) , \end{aligned}$$

where $P(x)= 1+x+x^2+ \dots + x^{\lambda -1}$. The above product equals 0 if and only if one of the following conditions holds:

- $\zeta _{q+1}^{e_1+qe'_1} -1 = 0$ $\iff$ $e_1+qe'_1 \equiv 0 \mod q+1$. That is, $e_1 \equiv e'_1 \mod q+1$; or
- $\left( \sum _{k=0}^{\frac{q-1}{2}} \zeta _{\frac{q+1}{2}}^{k(e_1 + q e'_1)}\right) =0$ $\iff$ $e_1+qe'_1 \not \equiv 0 \mod \frac{q+1}{2}$. Since $q\equiv -1 \mod \frac{q+1}{2}$, this is equivalent to $e_1 \not \equiv e'_1 \mod \frac{q+1}{2}$; or
- $P\left( \zeta _{\lambda (q+1)}^{(e_1+qe'_1)}\right) =0$. This is true if and only if $\zeta _{\lambda (q+1)}^{(e_1+qe'_1)}$ is a $\lambda$-th root of unity other than 1. That is equivalent to $e_1+qe'_1 \equiv 0 \mod q+1$ and $e_1+qe'_1\not \equiv 0 \mod \lambda (q+1)$, which is a particular case of the first condition.

Therefore, if either of the first two conditions hold, the sum (5) equals 0 and that implies that ${{\,\textrm{ev}\,}}_{{\varvec{v}}}(X^{{\varvec{e}}})$ and ${{\,\textrm{ev}\,}}_{{\varvec{v}}}(X^{\varvec{e'}})$ are orthogonal for the Hermitian inner product. $\square$

#### Remark 3.2

Consider the case when the twist vector is ${\varvec{1}}$, $\lambda =1$ and $A_j$ is the set of $q+1$-th roots of unity, that is the solutions to $X_j^{q+1}-1=0$, for every $j=1,\dots ,m$. Then for any $\Delta \subseteq E$ the GMCC $C_{{\varvec{1}},\Delta }$ is an Affine Variety Code (AVC) and it is not self-orthogonal (for the Hermitian inner product). This is because when we compute the Hermitian inner product of the evaluations of any monomial $X^{{\varvec{e}}}=X^{(e_1,\dots ,e_m)}$ with itself, one obtains that

$$\begin{aligned} {{\,\textrm{ev}\,}}_{{\varvec{1}}}(X^{{\varvec{e}}}) \cdot _h {{\,\textrm{ev}\,}}_{{\varvec{1}}}(X^{{\varvec{e}}})&= \sum _{\varvec{\alpha }\in E} \zeta _{q+1}^{\alpha _1e_1 (1+ q)} \zeta _{q+1}^{\alpha _2e_2 (1+ q)} \cdots \zeta _{q+1}^{\alpha _me_m (1+ q)}\\&= \left( \sum _{\alpha _1=0}^{q} \zeta _{q+1}^{\alpha _1e_1(1 + q)} \right) \left( \sum _{\alpha _2=0}^{q} \zeta _{q+1}^{\alpha _2e_2(1 +q)}\right) \dots \left( \sum _{\alpha _m=0}^{q} \zeta _{q+1}^{\alpha _me_m(1 + q)}\right) \end{aligned}$$

and every factor above is

$$\begin{aligned} \sum _{k=0}^q \zeta _{q+1}^{ke_1(1 + q)} =q+1 \ne 0. \end{aligned}$$

Thus, the evaluation of a monomial is not orthogonal to itself, and these codes are not self-orthogonal. However, we are able to provide a twist vector ${\varvec{v}}$ (3) to construct a self-orthogonal GMCC $C_{{\varvec{v}},\Delta }$ which is isometric to the non-self-orthogonal AVC $C_{{\varvec{1}},\Delta }$. The problem of not getting evaluations of monomials to be self-orthogonal can happen also with other twist vectors, that is why one has to choose the twist vector carefully.

### Our general construction

Before stating the theorem that is the general construction of this paper, recall the definition of the set *E* in the previous section. We define a subset in *E* which will be useful in the following.

#### Definition 3.3

Let $$E_0:=\left\{ {\varvec{e}}=(e_1,\dots ,e_m)\in E \mid 0 \le e_1\le \frac{q-1}{2}\right\} \subseteq E$$.

The next theorem shows that the set $E_0$ introduced in Definition 3.3 is used as a reference to construct Hermitian self-orthogonal GMCCs.

#### Theorem 3.4

Let *q* be an odd prime power and let $m\ge 1$, $\lambda \mid q-1$, $a_1:=\lambda (q+1)$ and $2\le a_j \le q^2-1$, $j=2,\dots ,m$ be positive integers. Let $n:=a_1\cdots a_m$. Consider the twist vector ${\varvec{v}}$ defined in (3) and the set $E_0\subseteq E$ introduced in Definition 3.3. Let $\Delta$ be a subset of $E_0$. Then,

$$\begin{aligned}C_{{\varvec{v}},\Delta }\subseteq (C_{{\varvec{v}},\Delta })^{\perp _h}.\end{aligned}$$

Therefore, there exists a stabilizer quantum code with parameters

$$\begin{aligned}{[}[n,n-2\#\Delta , \ge d]]_q\end{aligned}$$

where $$d={{\,\textrm{d}\,}}((C_{{\varvec{1}},\Delta })^{\perp _e})$$.

#### Proof

Since for all $(e_1,\dots ,e_m)\in \Delta$ we have $e_1\le \frac{q-1}{2}$, the self-orthogonality follows from Proposition 3.1. The existence and parameters of the stabilizer quantum code follow from Theorem 2.1. Notice that $d={{\,\textrm{d}\,}}((C_{{\varvec{v}},\Delta })^{\perp _h})$, but from Corollary 2.12 we can conclude that $d={{\,\textrm{d}\,}}((C_{{\varvec{1}},\Delta })^{\perp _e})$. $\square$

Notice that in the above theorem we do not give an explicit bound for the minimum distance, but it can be computed using Corollary 2.8 in every particular case.

### Our specific construction

Now we are going to provide a strategy [30] to choose a set $\Delta \subseteq E_0$ so that we can control the minimum distance ${{\,\textrm{d}\,}}((C_{{\varvec{1}},\Delta })^{\perp _e})$ and it maximizes the dimension of the resulting stabilizer quantum code. To that purpose, we need the following

#### Definition 3.5

Let $2\le t \le \frac{q+3}{2}$ be a positive integer. Define

$$\begin{aligned} \Delta _t:=\left\{ {\varvec{e}}=(e_1,\ldots ,e_m)\in E \ \big |\vert \ \prod _{j=1}^m (e_j+1)<t\right\} \subseteq E. \end{aligned}$$

**Fig. 2:**
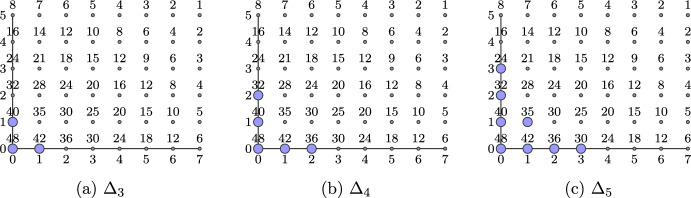
Sets $\Delta _3$, $\Delta _4$ and $\Delta _5$, where $m=2$, $a_1=8$ and $a_2=6$. We use the same conventions as in Fig. 1

Some instances of the above set are represented in Fig. 2.

#### Lemma 3.6

Let $\Delta _t\subseteq E$ be the set introduced in Definition 3.5. Then,

$$\begin{aligned}{{\,\textrm{d}\,}}\left( (C_{{\varvec{1}},\Delta _t})^{\perp _e}\right) \ge t.\end{aligned}$$

#### Proof

Using the notations in [25, Section 3], the authors define a code $C(L_2)$, where

$$\begin{aligned}L_2=\{X_1^{i_1}\cdots X_m^{i_m}\in \Delta (s_1,\dots ,s_m) \mid D^\perp (X_1^{i_1}\cdots X_m^{i_m})<\delta ^\perp \}.\end{aligned}$$

By choosing their $(s_1,\dots ,s_m)$ and $\delta ^\perp$ equal to our $(a_1,\dots ,a_m)$ and *t*, respectively, then we have that

$$\begin{aligned}L_2=\{X^{{\varvec{e}}} \mid {\varvec{e}}\in \Delta _t\},\end{aligned}$$

so $C(L_2)=C_{{\varvec{1}},\Delta _t}$, see [25, Definition 15]. The statement follows from their equation (8) in Section 3. $\square$

#### Theorem 3.7

Let *q* be an odd prime power and let $m\ge 1$, $\lambda \mid q-1$, $a_1:=\lambda (q+1)$ and $2\le a_j \le q^2-1$, $j=2,\dots ,m$ be positive integers. Let $n:=a_1\cdots a_m$. Consider the twist vector ${\varvec{v}}$ defined in (3), a positive integer

$$\begin{aligned}2\le t \le \frac{q+3}{2}\end{aligned}$$

and the set $\Delta _t\subseteq E$ introduced in Definition 3.5. Then, the following inclusion holds

$$\begin{aligned}C_{{\varvec{v}},\Delta _t}\subseteq (C_{{\varvec{v}},\Delta _t})^{\perp _h}.\end{aligned}$$

Therefore, there exists a stabilizer quantum code with parameters

$$\begin{aligned}{[}[n,n-2\#\Delta _t, \ge t]]_q.\end{aligned}$$

#### Proof

Let ${\varvec{e}}\in \Delta _t$. From $\prod _{j=1}^m (e_j+1)<t$, we have that $e_1< t-1$. Since $t \le \frac{q+3}{2}$, then $e_1< t-1\le \frac{q+1}{2}$ and therefore $\Delta _t\subseteq E_0$. So, from Theorem 3.4 we have that $C_{{\varvec{v}},\Delta _t}\subseteq (C_{{\varvec{v}},\Delta _t})^{\perp _h}$.

The existence and parameters of the stabilizer quantum code follows from Theorem 2.1. Notice that from Corollary 2.12 and Lemma 3.6, we have ${{\,\textrm{d}\,}}((C_{{\varvec{v}},\Delta _t})^{\perp _h})={{\,\textrm{d}\,}}((C_{{\varvec{1}},\Delta _t})^{\perp _e})\ge t$. $\square$

### The dimension

We state a recursive formula for the dimension of the quantum code, which is shown in [30].

Let $a, b\in {{\mathbb {N}}}$. Consider the case when $a_j=b$ for all $j=1, \dots , m$. We define

$$\begin{aligned} V_b(m,a):=\# \left\{ (l_1, \dots , l_m) \ \big |\vert \ l_j \in {{\mathbb {N}}}, \ 1\le l_j \le b, \ j = 1, \dots m, \ \prod _{j=1}^m l_j \le a\right\} . \end{aligned}$$

In [30], they give the following recursive formula:

$$\begin{aligned} V_b(m,a)=\sum _{s=1}^{b}V\left( m-1, \left\lfloor {\frac{a}{s}}\right\rfloor \right) , \end{aligned}$$

where $$V_b(1,a)= \min \{a,b\}$$.

Observe that $\#\Delta _t = V_{\lambda (q+1)}(m,t-1)$, where all of $a_1, \ldots ,a_m$ are equal to $\lambda (q+1)$. Therefore, we can use the recursive formula described above to compute $\#\Delta _t$, and hence the dimension of the quantum code in Theorem 3.7. For example, when $m=2$

6 $$\begin{aligned} \#\Delta _t=V_{\lambda (q+1)}(2,t-1)=t-1+\left\lfloor {\frac{t-1}{2}}\right\rfloor +\left\lfloor {\frac{t-1}{3}}\right\rfloor +\cdots +\left\lfloor {\frac{t-1}{t-2}}\right\rfloor + \left\lfloor {\frac{t-1}{t-1}}\right\rfloor , \end{aligned}$$

and when $$m=3$$

$$\begin{aligned}\#\Delta _t=V_{\lambda (q+1)}(3,t-1)=\sum _{\alpha =1}^{t-1} \sum _{\beta =1}^{\left\lfloor {\frac{t-1}{\alpha }}\right\rfloor }\left\lfloor {\frac{t-1}{\alpha \beta }}\right\rfloor .\end{aligned}$$

## We obtain MDS and Hermitian almost MDS quantum codes

In this section, we prove that we can obtain quantum codes that are close to the singleton bound. Let us recall first the quantum singleton bound.

### Lemma 4.1

(Quantum Singleton bound [48]) If a stabilizer quantum code with parameters $[[n,k,d]]_q$ exists, then $n\ge k+2d-2$.

Codes attaining equality are called quantum MDS codes.

### MDS

#### Theorem 4.2

The stabilizer quantum codes obtained from Theorem 3.7 with $m=1$ are quantum MDS codes.

#### Proof

For any given bound for the minimum distance $t\in \{2,\dots , \frac{q+3}{2}\}$, we have $\Delta _t=\{0,1,2,\ldots ,t-2\}$. The parameters of the stabilizer quantum code constructed from Theorem 3.7 are:

$$\begin{aligned} {[}[n,k,d]]_q=[[\lambda (q+1),\lambda (q+1) - 2(t-1),\ge t]]_q. \end{aligned}$$

It is easily verified that the above parameters provide a quantum MDS code, because $k+2d\ge \lambda (q+1) - 2(t-1)+2t=\lambda (q+1) +2=n+2$ and the quantum singleton bound gives an equality. $\square$

Some sample parameters are given in Tables 3, 4, 5, 6, 7. For example, we obtain quantum MDS codes with parameters $[[12,8,3]]_5$ in Table 4, $[[8,4,3]]_7$ and $[[16,8,5]]_7$ in Table 5 and $[[20,12,5]]_9$ in Table 6. We do not claim that these examples are new.

The article [54] recently appeared on the arxiv and has a construction of MDS codes with lengths of the form $r(q^2-1)/h$ where *h* is an even divisor of $q-1$ and $r\le h/2$ (their Theorems 3, 4 and 5). This article does not provide an explicit twist vector (they prove the existence of it). Our construction has an explicit twist vector and (in the $m=1$ case) gives codes with the same parameters.

### Hermitian almost MDS

The quantum singleton defect of a parameter set *n*, *k*, *d* is defined to be $n-(k+2d-2)$. MDS codes have quantum singleton defect 0, by definition. Codes with quantum Singleton defect 1 are called quantum almost MDS (QAMDS) codes. However, from the statement of Theorem 2.1, one can see that the quantum singleton defect of any code constructed using Theorem 2.1 must be even, and thus, a quantum singleton defect of 1 cannot be achieved. The smallest nonzero singleton defect of a code constructed using Theorem 2.1 is therefore 2. This motivates the following definition.

#### Definition 4.3

A quantum code constructed from Theorem 2.1 with parameters $[[n,k,d]]_q$ such that $n=k+2d$ is called a *quantum Hermitian almost MDS* (*QHAMDS*) code.

In Theorem 4.2, we showed that we can construct quantum MDS codes. Recall that the quantum MDS conjecture [35] states that $n\le q^2+1$ for a quantum MDS code with parameters $[[n,k,d]]_q$ and *q* odd. Now we are going to show that we can also construct quantum codes with $n>q^2+1$ that are at least QHAMDS. That is, they are either QHAMDS or MDS. If the quantum MDS conjecture is true, they cannot be MDS, and therefore they would have the best possible parameters.

#### Theorem 4.4

The stabilizer quantum codes obtained from Theorem 3.7 with $m=2$, $n>q^2+1$ and $t=3$ are at least QHAMDS.

#### Proof

Let $m=2$, $t=3$ and $\lambda$ and $a_2$ be as defined in Theorem 3.7 such that $n>q^2+1$. We have $\Delta _3=\{(0,0),(1,0),(0,1)\}$ (see Fig. 2). The parameters of the stabilizer quantum code constructed from Theorem 3.7 are

$$\begin{aligned} {[}[n,k,d]]_q=[[\lambda (q+1)a_2,\lambda (q+1)a_2 - 6,\ge 3]]_q. \end{aligned}$$

It is easily verified that the above parameters provide a code which is at least QHAMDS. This is because $k+2d\ge \lambda (q+1)a_2 - 6+2\cdot 3=\lambda (q+1)a_2 =n$. $\square$

Some examples will be given in Tables 3 to 7. In [16], the authors study ternary quantum codes of minimum distance three. In that paper (their Theorem 4.4), quantum codes with parameters $[[n,n-7,3]]_3$ are shown for certain lengths *n*. For those lengths which are a multiple of 4 and less than 64, we can improve the dimension by 1, using the codes in Theorem 4.4. See also Table 3.

## When $m=2$ we can beat Gilbert–Varshamov bound

In this section, we include a proof that an infinite family of codes obtained from our constructions will beat the quantum Gilbert–Varshamov bound when $m=2$. We remark that the codes with $m>2$ can also beat the Gilbert–Varshamov bound, some examples when $m=3$ are presented in Tables 3, 4 and 6.

Let us recall the quantum Gilbert–Varshamov bound whose proof can be found in [22]:

### Theorem 5.1

(Quantum Gilbert–Varshamov Bound) Suppose that $n>k \ge 2$, $d\ge 2$, and $n\equiv k \mod 2$. If

7 $$\begin{aligned} \frac{q^{n-k+2}-1}{q^2-1}\ge \sum _{i=1}^{d-1} (q^2-1)^{i-1} {n \atopwithdelims ()i} \end{aligned}$$

then there exists a pure stabilizer quantum code with parameters $[[n,k,d]]_q$.

We say that a parameter set *n*, *k*, *d*, *q* beats the QGV bound if the inequality (7) is not satisfied.

In the $m=2$ case, we have the following statement, using the codes constructed in this paper. In this statement, we are using the formula (6).

### Theorem 5.2

Given an odd prime power *q*, and given *d* in the range $5\le d \le (q+3)/2$, let *n* be in the interval

$$\begin{aligned} \biggl ((d-1)^{d-1} \frac{q^2}{(q^2-1)^{d-1}} q^{2(d-1) (0.7+\ln (d-1))}\biggr )^{\frac{1}{d-1}} \le n \le (q^2-1)^2 \end{aligned}$$

and have the form $\lambda (q+1)a_2$ where $\lambda \mid (q-1)$ and $2\le a_2 \le q^2-1$. Then, there exists a quantum code with parameters

$$\begin{aligned}{[}[n,n-2\sum _{j=1}^{d-1} \left\lfloor {\frac{d-1}{j}}\right\rfloor , \ge d]]_q\end{aligned}$$

and this code beats the quantum Gilbert–Varshamov bound.

### Proof

We use the codes whose existence is proved in Theorem 3.7 in the case $m=2$. The upper bounds $d\le (q+3)/2$ and $n \le (q^2-1)^2$ follow from the construction in Theorem 3.7.

Let

$$\begin{aligned} A=\sum _{i=1}^{d-1} (q^2-1)^{i-1} {n \atopwithdelims ()i} \end{aligned}$$

and let

$$\begin{aligned} D= \frac{q^{n-k+2}-1}{q^2-1} \end{aligned}$$

where $k=n-2\sum _{j=1}^{d-1} \left\lfloor {\frac{d-1}{j}}\right\rfloor$ (this dimension formula comes from (6) which uses our construction with $m=2$). We wish to prove that $A>D$ under the stated hypotheses. To prove this, we are going to let

$$\begin{aligned} B=\frac{1}{(d-1)^{d-1}} n^{d-1} (q^2-1)^{d-2} \end{aligned}$$

and let

$$\begin{aligned} C=\biggl ( \frac{q^2}{q^2-1} \biggr ) \ q^{2(d-1) (0.7 + \ln (d-1))} \end{aligned}$$

and we will prove three things: that $A>B$, that $B\ge C$, and that $C>D$. This will complete the proof that $A>D$.

To show that $A>B$, we will use the estimate for binomial coefficients ${n \atopwithdelims ()k} > (\frac{n}{k})^k$. Then

$$\begin{aligned} A=\sum _{i=1}^{d-1} (q^2-1)^{i-1} {n \atopwithdelims ()i}&> {n \atopwithdelims ()d-1} (q^2-1)^{d-2}\\&> \biggl ( \frac{n}{d-1}\biggr )^{d-1} (q^2-1)^{d-2}\\&=\frac{1}{(d-1)^{d-1}} n^{d-1} (q^2-1)^{d-2}=B. \end{aligned}$$

To prove that $B\ge C$, rearranging the hypothesis

$$\begin{aligned} \biggl ((d-1)^{d-1} \frac{q^2}{(q^2-1)^{d-1}} q^{2(d-1) (0.7+\ln (d-1))}\biggr )^{\frac{1}{d-1}} \le n \end{aligned}$$

yields precisely that $B\ge C$.

To prove that $C>D$, we will use the fact that if $r\ge 4$ then $H_r < 0.7+\ln r$ where $H_r$ is the *r*-th harmonic number defined by $H_r = \sum _{j=1}^r \frac{1}{j}$. Then,

$$\begin{aligned} \sum _{j=1}^{d-1} \left\lfloor {\frac{d-1}{j}}\right\rfloor&< \sum _{j=1}^{d-1} \frac{d-1}{j} \\&=(d-1) H_{d-1}\\&< (d-1) (0.7 + \ln (d-1)) \qquad \text {since }d-1\ge 4. \end{aligned}$$

It follows that

$$\begin{aligned} D&=\frac{q^{n-k+2}-1}{q^2-1} \\&< \frac{q^{n-k+2}}{q^2-1} \\&= \biggl ( \frac{q^2}{q^2-1} \biggr ) \ q^{n-k}\\&= \biggl ( \frac{q^2}{q^2-1} \biggr ) \ q^{2\sum _{j=1}^{d-1} \left\lfloor {\frac{d-1}{j}}\right\rfloor }\\&< \biggl ( \frac{q^2}{q^2-1} \biggr ) \ q^{2(d-1) (0.7 + \ln (d-1))}=C. \end{aligned}$$

$$\square $$

In this theorem, we assumed that $d\ge 5$ because of the constant 0.7, which is a choice. The cases $d=3$ and $d=4$ can be proved separately. They could be included in the proof above but the constant 0.7 would have to be larger. Similarly, we could have stated the theorem for $d\ge 6$ and the constant would be smaller, it would be 0.68. Then, the $d=5$ case would need to be handled separately. As *d* gets larger, the constant gets smaller and approaches the Euler–Mascheroni constant.

We show Table 1 where for each *q* between 7 and 17 and $d=5,6,7$ we give the range of values of *n* for which the quantum Gilbert–Varshamov bound is beaten, as given by Theorem 5.2.

**Table 1:** Some instances of the range of lengths of codes (from Theorem 5.2 only) that beat the quantum Gilbert–Varshamov bound

*d*	*q*				
	7	9	11	13	17
5	742-2304	1438-6400	2450-14400	3818-28224	7800-82944
6	$d>\frac{q+3}{2}$	3848-6400	7022-14400	11600-28224	26006-82944
7	$d>\frac{q+3}{2}$	$d>\frac{q+3}{2}$	None	None	72590-82944

A separate special analysis for each *d*, or using better estimates in the proof, or using a computer, will give a better range of values for *n* than the statement of Theorem 5.2. For example, when $q=7$ and $d=5$, computer calculations show that the Gilbert–Varshamov bound is beaten by our codes as soon as $n>295$, whereas the proof of Theorem 5.2 gives $n\ge 742$. As another example, when $q=11$ and $d=7$, the range of values of *n* as given by the statement of Theorem 5.2 is empty (in the table we wrote ‘none’). However, there are in fact values of *n* that beat the Gilbert–Varshamov bound. We state one example $[[7200,7172,7]]_{11}$ in Table 7.

We also remark that Theorem 5.2 is for $m=2$. A similar result will hold for $m>2$.

### $d=3$

In the previous theorem, we assumed that $d\ge 5$ to obtain a slightly stronger statement. We will treat the case that $d=3$ (and $m=2$) separately, and we will complete the analysis in detail now. We omit the $d=4$ case, which is similar.

Suppose $d=3$. By the formula (6) we have that $\Delta _3$ has 3 elements, see also Fig. 2. The two sides of the Gilbert–Varshamov bound become

$$\begin{aligned} \frac{q^{n-k+2}-1}{q^2-1}= \frac{q^{8}-1}{q^2-1} =q^6+q^4+q^2+1\end{aligned}$$

and

$$\begin{aligned} \sum _{i=1}^{d-1} (q^2-1)^{i-1} {n \atopwithdelims ()i}= n+{n \atopwithdelims ()2}(q^2-1). \end{aligned}$$

To beat the G–V bound, we obtain a condition which is a quadratic polynomial in *n*, namely we require that

$$\begin{aligned} n+{n \atopwithdelims ()2}(q^2-1) - (q^6+q^4+q^2+1)>0. \end{aligned}$$

Solving the quadratic yields that the G–V bound is beaten when

$$\begin{aligned} n>\frac{q^2-3+\sqrt{8q^8+q^4-6q^2+1}}{2(q^2-1)}. \end{aligned}$$

For $m=2$ the largest possible *n* is $(q-1)(q+1)(q^2-1)$. Therefore, for each valid *n* which is a multiple of $q+1$ between $\frac{q^2-3+\sqrt{8q^8+q^4-6q^2+1}}{2(q^2-1)}$ and $(q^2-1)^2$ we obtain a code of that length that beats the G–V bound.

We show Table 2 where for each *q* and $d=3$ we state the range of values of *n* for which Gilbert–Varshamov bound is beaten.

**Table 2:** Some instances of the range of lengths of codes from Theorem 3.7 with $d=3$ that beat the quantum Gilbert–Varshamov bound

*q*	3	5	7	9	11
Range of lengths	15-64	38-576	72-2304	117-6400	174-14400

In the $d=4$ case (details omitted), the polynomial in *n* would be cubic instead of quadratic.

**Table 3:** A $q=3$ sample of codes

*m*	$a_1$	$a_2$	$a_3$	Quantum code	Beats QGV	Comment
1	4			$[[4,0,3]]_3$	No	MDS
1	8			$[[8,4,3]]_3$	Yes	MDS
2	4	5		$[[20,14,3]]_3$	Yes	QHAMDS
2	4	6		$[[24,18,3]]_3$	Yes	QHAMDS
2	4	7		$[[28,22,3]]_3$	Yes	QHAMDS
2	4	8		$[[32,26,3]]_3$	Yes	QHAMDS, equals $[[32,26,3]]_3$ in [16]
2	8	5		$[[40,34,3]]_3$	Yes	QHAMDS, beats $[[40,33,3]]_3$ in [16]
2	8	6		$[[48,42,3]]_3$	Yes	QHAMDS, equals $[[48,42,3]]_3$ in [16]
2	8	7		$[[56,50,3]]_3$	Yes	QHAMDS, beats $[[56,49,3]]_3$ in [16]
2	8	8		$[[64,58,3]]_3$	Yes	QHAMDS, beats $[[64,57,3]]_3$ in [16]
3	8	3	3	$[[72,64,3]]_3$	Yes	Beats $[[72,62,3]]_3$ in [39]
3	4	8	4	$[[128,120,3]]_3$	Yes	Length not obtained with $m=1, 2$

**Table 4:** A $q=5$ sample of codes

*m*	$a_1$	$a_2$	$a_3$	Quantum Code	Beats QGV	Comment
1	6			$[[6,2,3]]_5$	No	MDS
1	12			$[[12,8,3]]_5$	Yes	MDS
1	12			$[[12,6,4]]_5$	Yes	MDS
2	6	5		$[[30,24,3]]_5$	No	QHAMDS, beats $[[33,13,3]]_5$ in [8]
2	6	6		$[[36,30,3]]_5$	No	QHAMDS
2	6	6		$[[36,26,4]]_5$	No	Length not obtained with $m=1$
2	6	7		$[[42,36,3]]_5$	Yes	QHAMDS
2	6	13		$[[78,72,3]]_5$	Yes	QHAMDS, beats $[[80,68,3]]_5$ in [8]
2	6	13		$[[78,68,4]]_5$	Yes	Beats $[[78,60,4]]_5$ in [43]
2	6	16		$[[96,86,4]]_5$	Yes	Same as in [43]
2	6	19		$[[114,104,4]]_5$	Yes	Length not obtained with $m=1$
2	6	22		$[[132,122,4]]_5$	Yes	Beats $[[132,118,4]]_5$ in [55]
2	12	24		$[[288,282,3]]_5$	Yes	QHAMDS
2	12	24		$[[288,278,4]]_5$	Yes	Beats $[[288,275,4]]_5$ in [28]
3	24	13	2	$[[624,612,4]]_5$	Yes	Same as in [28]
3	24	24	2	$[[1152,1144,3]]_5$	Yes	Length not obtained with $m=1, 2$

**Table 5:** A $q=7$ sample of codes

*m*	$a_1$	$a_2$	$a_3$	Quantum Code	Beats QGV	Comment
1	8			$[[8,4,3]]_7$	No	MDS
1	16			$[[16,12,3]]_7$	Yes	MDS
1	16			$[[16,10,4]]_7$	Yes	MDS
1	16			$[[16,8,5]]_7$	Yes	MDS
1	24			$[[24,20,3]]_7$	Yes	MDS, same as [54]
1	48			$[[48,44,3]]_7$	Yes	MDS
2	8	7		$[[56,50,3]]_7$	No	QHAMDS
2	8	8		$[[64,58,3]]_7$	No	QHAMDS, beats $[[65,53,3]]_7$ in [38]
2	8	8		$[[64,54,4]]_7$	No	Length not obtained with $m=1$
2	8	8		$[[64,48,5]]_7$	No	Beats $[[65,41,5]]_7$ in [38]
2	8	9		$[[72,66,3]]_7$	Yes	QHAMDS, beats $[[75,63,3]]_7$ in [43]
2	8	9		$[[72,56,5]]_7$	No	Beats $[[75,51,5]]_7$ in [43]
2	8	15		$[[120,114,3]]_7$	Yes	QHAMDS, beats $[[126,114,3]]_7$ in [8]
2	8	21		$[[168,162,3]]_7$	Yes	QHAMDS, beats $[[168,158,3]]_7$ in [8]
2	8	21		$[[168,158,4]]_7$	Yes	Beats $[[168,152,4]]_7$ in [8]
2	8	25		$[[200,190,4]]_7$	Yes	Same as in [43]
2	8	48		$[[384,378,3]]_7$	Yes	QHAMDS, same as in [14]
2	8	48		$[[384,374,4]]_7$	Yes	Same as in [14]
2	8	48		$[[384,368,5]]_7$	Yes	Same as in [14]
2	16	27		$[[432,422,4]]_7$	Yes	Beats $[[432,419,4]]_7$ in [28]
3	16	48	2	$[[768,760,3]]_7$	Yes	Length not obtained with $m=1, 2$

**Table 6:** A $q=9$ sample of codes

*m*	$a_1$	$a_2$	$a_3$	Quantum Code	Beats QGV	Comment
1	10			$[[10,6,3]]_9$	No	MDS
1	20			$[[20,16,3]]_9$	Yes	MDS
1	20			$[[20,14,4]]_9$	Yes	MDS
1	20			$[[20,12,5]]_9$	Yes	MDS
1	40			$[[40,36,3]]_9$	Yes	MDS
2	10	10		$[[100,80,6]]_9$	Yes	Length not obtained with $m=1$
2	10	24		$[[240,230,4]]_9$	Yes	Beats $[[246,228,4]]_9$ in [43]
2	10	55		$[[550,534,5]]_9$	Yes	Length not obtained with $m=1$
3	80	80	2	$[[12800,12792,3]]_9$	Yes	Length not obtained with $m=1,2$

**Table 7:** A $q=11$ sample of codes

*m*	$a_1$	$a_2$	$a_3$	Quantum Code	Beats QGV	Comment
1	12			$[[12,8,3]]_{11}$	No	MDS
1	12			$[[12,6,4]]_{11}$	Yes	MDS
1	12			$[[12,4,5]]_{11}$	Yes	MDS
1	60			$[[60,56,3]]_{11}$	Yes	MDS
1	60			$[[60,54,4]]_{11}$	Yes	MDS
1	60			$[[60,52,5]]_{11}$	Yes	MDS
2	12	15		$[[180,174,3]]_{11}$	Yes	QHAMDS, beats $[[183,171,3]]_{11}$ in [43]
2	12	15		$[[180,164,5]]_{11}$	No	Beats $[[183,159,5]]_{11}$ in [43]
2	60	120		$[[7200,7172,7]]_{11}$	Yes	Length not obtained with $m=1$

## Examples

Tables 3, 4, 5, 6, 7 show some samples of small values of the parameters of the quantum codes constructed with Theorem 3.7. For their minimum distance, we give the lower bound *t* provided by Theorem 3.7. We remind the reader of our notation: *q* is an odd prime power, $a_1$ can be any $\lambda (q+1)$ where $\lambda$ is a divisor of ${q-1}$, and $a_2$ and $a_3$ can take any value between 2 and $q^2-1$.

Note that for codes $[[n,k,d]]_q=[[n,k,\ge t]]_q$ constructed from Theorem 3.7 we must have $t\le \frac{q+3}{2}=3$ when $q=3$, and $t\le \frac{q+3}{2}=4$ when $q=5$.

Recall also codes with $n+2=k+2d$ are called MDS codes and codes with $n=k+2d$ are called QHAMDS codes. We also say in the sixth column if that code beats the quantum Gilbert–Varshamov bound in the sense explained before Theorem 5.2.

In order to compare different quantum codes one may use the *length extension*, *subcode* and *smaller distance* propagation rules, as stated in [44] for example. We therefore say that a quantum $[[n,k,d]]_q$ code beats a quantum $[[n',k',d']]_q$ code if at least one of the following holds:

- $n<n'$ and $k=k'$ and $d=d'$ (*length extension*)
- $n=n'$ and $k>k'$ and $d=d'$ (*subcode*)
- $n=n'$ and $k=k'$ and $d>d'$. (*smaller distance*)

In other words, decreasing *n*, or increasing *k*, or increasing *d*, while keeping other parameters fixed, results in a better code. This is well known, see [44] for example, where the authors say that “...all other parameters being equal, we record the smallest *n*, the largest *k*, the largest *d*,...”.

In the tables below we give some examples of codes that result from our construction, and compare them to the best known codes in the literature. In some cases, we improve on the best known.

It is possible to have more than one improvement. For example, a $[[78,72,3]]_5$ code beats a $[[80,68,3]]_5$ code in two ways, because it has a smaller *n* and also has a larger *k*.

Finally, the article [54] recently appeared on the arxiv and has a construction of MDS codes with lengths of the form $r(q^2-1)/h$ where *h* is an even divisor of $q-1$ and $r\le h/2$ (their Theorems 3, 4 and 5). Some of the MDS codes appearing in our tables may also be obtained with their construction.

## Data Availability

Data sharing is not applicable to this article as no datasets were generated or analysed during the current study.
